# Loss of plasticity in maturation timing after ten years of captive spawning in a delta smelt conservation hatchery

**DOI:** 10.1111/eva.13611

**Published:** 2023-11-02

**Authors:** Melanie E. F. LaCava, Joanna S. Griffiths, Luke Ellison, Evan W. Carson, Tien‐Chieh Hung, Amanda J. Finger

**Affiliations:** ^1^ Genomic Variation Laboratory, Department of Animal Science University of California, Davis Davis California USA; ^2^ Department of Environmental Toxicology and Department of Wildlife, Fish, and Conservation Biology University of California, Davis Davis California USA; ^3^ Fish Conservation and Culture Laboratory, Department of Biological and Agricultural Engineering University of California, Davis Davis California USA; ^4^ US Fish and Wildlife Service San Francisco Bay‐Delta Fish and Wildlife Office Sacramento California USA

**Keywords:** age at maturity, conservation, delta smelt, domestication selection, heritability, phenotypic plasticity, relaxed selection

## Abstract

Adaptation to captivity in spawning programs can lead to unintentional consequences, such as domestication that results in reduced fitness in the wild. The timing of sexual maturation has been shown to be a trait under domestication selection in fish hatcheries, which affects a fish's access to mating opportunities and aligning their offspring's development with favorable environmental conditions. Earlier maturing fish may be favored in hatchery settings where managers provide artificially optimal growing conditions, but early maturation may reduce fitness in the wild if, for example, there is a mismatch between timing of reproduction and availability of resources that support recruitment. We investigated patterns of maturation timing in a delta smelt (*Hypomesus transpacificus*) conservation hatchery by quantifying changes to the median age at maturity since the captive spawning program was initiated in 2008. Over the span of a decade, we observed a small, but significant increase in age at maturity among broodstock by 2.2 weeks. This trait had low heritability and was largely controlled by phenotypic plasticity that was dependent on the time of year fish were born. Fish that were born later in the year matured faster, potentially a carryover from selection favoring synchronous spawning in the wild. However, higher DI (domestication index) fish showed a loss of plasticity, we argue, as a result of hatchery practices that breed individuals past peak periods of female ripeness. Our findings suggest that the hatchery setting has relaxed selection pressures for fish to mature quickly at the end of the year and, consequently, has led to a loss of plasticity in age at maturity. Hatchery fish that are re‐introduced in the wild may not be able to align maturation with population peaks if their maturation rates are too slow with reduced plasticity, potentially resulting in lower fitness.

## 1 INTRODUCTION

Captive breeding programs aid in the conservation of critically endangered species by serving as a refuge population to prevent extinction, by producing individuals for supplementation or reintroduction, and by providing opportunities for research (Allendorf et al., 2012; Fisch et al., 2013, 2015; Frankham, 2008; Frankham et al., 1986; Naish et al., 2007; Pollard & Flagg, 2004). In cultivating a captive population, a primary goal is to minimize adverse effects of collection, mating, and rearing practices in order to maintain phenotypic and genetic similarity to the wild population (Fisch et al., 2015; Russello & Jensen, 2018). Bringing a relatively small number of individuals into artificial conditions for captive breeding can lead to unintentional genetic effects such as inbreeding, genetic drift, and adaptation to captivity which can lead to a loss of genetic diversity and compromise fitness in the wild (Laikre et al., 2010; Lorenzen et al., 2012).

Trait changes associated with hatcheries can occur through two non‐mutually exclusive mechanisms: (1) domestication selection in which traits that increase survival and reproductive fitness are favored in captive conditions but where “wild” traits may be disadvantageous (directional selection/adaptation to captivity) and (2) relaxed selection where natural selection pressures in the wild are absent in captive environments (McDermid et al., 2010). Controlled hatchery environments often reduce selection pressures associated with competition for resources, disease resistance, and predator avoidance while also introducing new selection pressures associated with maximizing survival and reproduction (Lorenzen et al., 2012; Thorpe, 2004). While these trait changes may enhance fitness in the hatchery environment, these altered traits are often maladaptive in the wild (Frankham, 2008). In hatchery‐born steelhead, for example, domestication resulted in faster growth rates, but this trait led to significantly lower survival in the wild (Blouin et al., 2021). In extreme cases, negative fitness consequences in the wild can arise after a single generation of captive spawning (Araki et al., 2008; Christie et al., 2012, 2014; Milot et al., 2013). Relaxed selection in a hatchery can also result in fish maladapted to the wild because traits selected against in the wild can increase in frequency in a hatchery where those negative selection pressures are relaxed or absent. For example, relaxed selection can occur when hatcheries employ random mating strategies for species that have sexual selection in the wild (Fisch et al., 2015; Thériault et al., 2011). In natural populations of Atlantic salmon, *Salmo salar*, females typically mate with males that will increase their offspring's diversity in the major histocompatibility complex (MHC) suite of genes, which has been shown to increase immune fitness by decreasing parasite loads (Consuegra & Garcia de Leaniz, 2008). However, a random mating strategy in a hatchery can remove this selection pressure by providing breeding opportunities for all fish, resulting in offspring that had higher parasite loads than free‐mating salmon (Consuegra & Garcia de Leaniz, 2008).

Adaptation to captivity can affect the age at which a fish reaches sexual maturity, with potential consequences for fitness and recruitment success for species that inhabit highly seasonal environments. The timing of sexual maturation and reproduction usually coincides with conditions that are favorable for offspring survival, such as abundant prey and food sources (Shuter et al., 2012) and low predator densities (Sancho et al., 2000). Additionally, the ability to alter maturation timing to align with synchronous reproduction increases the probability of finding a suitable mate (Rowe & Hutchings, 2003). Age at maturity is often plastic in response to environmental conditions such as rearing temperature (Moyle & Cech, 2004) and diet quantity and quality (Jonsson et al., 2012; Larsen et al., 2006), but can also have heritable components that selection can act on. In Chinook salmon, for example, age at maturity is partially genetically determined and a shift toward earlier male maturity in a hatchery was identified as a mechanism of adaptation to captivity in this species (Ford et al., 2012), leading to reduced spawning success in the wild (Ford et al., 2015).

Although genetic adaptation to captivity, including changes to maturation timing, has been extensively studied in salmonids, little is known about the consequences of changes to maturation timing in conservation hatcheries of other critically endangered fish species, such as the delta smelt, *Hypomesus transpacificus*. The delta smelt is a small, osmerid species, which may be more susceptible to adaptation to captivity because of its predominantly annual reproductive life cycle spent entirely in the hatchery (Finger et al., 2018). The delta smelt conservation hatchery is genetically managed by minimizing relatedness of spawners, equalizing family sizes, and incorporating wild fish every year with the goal of preserving a captive refuge population that is as representative of the wild population as possible (Fisch et al., 2013; Lindberg et al., 2013). Despite this intensive genetic management, adaptation to captivity has been documented: fish from families that had spent more generations in captivity (i.e., higher domestication index, DI) produced more offspring that survived to maturity the following year (Finger et al., 2018). Finger et al. (2018) noted that the time of year when fish became mature influenced their inclusion in the spawning program, with potential implications for fitness. The hatchery begins spawning fish when they start to sexually mature (usually in January) and ends the spawning season once a target number of pairs have been successfully mated (usually in May). Surveys suggest that in the wild the spawning season typically lasts from January to June (Moyle et al., 2016). Fish that become mature outside of the hatchery spawning period will not be included in the spawning program, resulting in a potential selection pressure against late maturing fish. This husbandry strategy risks selection for early maturation as these individuals have a higher probability of being spawned. As a result, a shift to earlier age at maturity could occur over time in the delta smelt hatchery population, similar to the increase in early maturation rates observed in a Chinook salmon hatchery (Ford et al., 2012).

Our goals were to characterize changes to age at maturity in a captive refuge population of delta smelt and evaluate whether this trait change explains the previously documented adaptation to captivity (i.e., high fitness of higher DI delta smelt; Finger et al., 2018). We used a decade of spawning season data and a complete genetic pedigree to first investigate whether there has been a shift in age at maturity over time. We then estimated the genetic (i.e., DI) and plastic (i.e., temperature and Julian week born) components of age at maturity to understand how these factors have contributed to changes in this trait. Finally, we examined whether this trait is undergoing selection in the hatchery by measuring fitness associated with changes in the median age of maturity and changes in the plasticity of age at maturity. Understanding the mechanisms of adaptation to captivity with over a decade of captive spawning data will help inform hatchery management practice for delta smelt and address the urgent need to preserve standing genetic variation as this species is nearly extinct in the wild.

## 2 METHODS

### 2.1 Captive spawning program

The delta smelt is endemic to the upper San Francisco Estuary in California, USA, and population declines since the 1980s (Moyle et al., 2016) led to its listing as threatened under the federal Endangered Species Act in 1993 (U.S. Fish and Wildlife Service, 1993) and endangered under the California Endangered Species Act in 2009 (California Department of Fish and Wildlife, 2009). In 2008, the University of California, Davis Fish Conservation and Culture Laboratory (FCCL), established a captive refuge population of delta smelt to prevent species extinction and produce fish for research (Lindberg et al., 2013). Delta smelt are an annual spawning fish with a life spawn of approximately one to  two years. Each spawning season (approximately mid‐January to mid‐May), the FCCL staff identified mature fish (i.e., gametes expressed after mild pressure applied to the abdomen), marked each with a unique tag, and collected a fin clip from each for genetic analysis and pedigree assignment. The FCCL sorted through tagged fish twice per week to identify ripe females (i.e., ready to spawn). Once a suitable genetic match was identified for the selected ripe females (see *Genetic management of spawning*), the FCCL conducted strip spawning by combining manually expressed eggs from a ripe female with manually expressed milt from a mature male (Ellison et al., 2023). Approximately equal numbers of fertilized eggs from eight single‐pair crosses were then combined into one incubator for hatching, creating a multi‐family group. Spawning continued throughout the season until 30+ multi‐family groups were created. See Lindberg et al. (2013) for detailed hatchery management practices.

Fish experienced two temperature regimes at the FCCL, depending on hatch date (Figure S1). All fertilized eggs were incubated at 16.5°C and juveniles were kept at this temperature until late autumn when the temperature was lowered to 12°C to reduce stress from handling during tank transfers and for transportation of a subset of the population to a second facility as a backup. Because fish were spawned throughout a 3.5‐month window each spring, fish born early in the season (typically crosses made from January to mid‐April, hereafter referred to as regular season fish) experienced the higher rearing temperature (16.5°C) for weeks or even months longer than fish born late in the season (typically crosses made after mid‐April, hereafter referred to as late season fish). After late autumn handling each year, the FCCL increased the rearing temperature of late season fish back to 16.5°C to allow them to experience the higher temperature for approximately the same total time as early season fish. Temperature can affect the growth rate of fish (Moyle & Cech, 2004), so the variation in temperature regime may have influenced ages at maturity. To avoid conflating our results with differences due to temperature regime, some analyses used only regular season fish (and excluded late season fish), as specified below.

### 2.2 Genetic management of spawning

Each year when mature fish were tagged prior to spawning, fin clips were collected and sent for parentage analysis by the University of California, Davis Genomic Variation Laboratory (GVL). From 2008 to 2019, the GVL genotyped fish at 12 microsatellite loci (Fisch et al., 2013), then in 2020 transitioned to a panel of 75 single‐nucleotide polymorphism (SNP) loci, after confirming concordance in parentage assignment between the two marker types (Lew et al., 2015). Each year, the GVL used genotype data from newly mature fish and from putative parents (i.e., fish spawned in the previous year) to perform genetic parentage assignment with CERVUS (Kalinowski et al., 2007) or COLONY (Jones & Wang, 2010) and reconstructed a pedigree with PMx (Lacy et al., 2012). See Finger et al. (2018) and Fisch et al. (2013) for detailed parentage analysis methods. Based on the reconstructed pedigree of tagged fish, the GVL identified suitable tagged males to pair with ripe females throughout the spawning season. Spawning pairs were selected based on several criteria: prioritizing the inclusion of wild fish, minimizing inbreeding calculated in PMx, and equalizing representation among families (Fisch et al., 2013).

### 2.3 Identifying changes in age at maturity over time

We calculated age at maturity as the number of weeks from fertilization to the date a fish was tagged as mature. We excluded age at maturity data from 2011 because in that year fish were tagged without regard to sexual maturity, so our analyses include the years 2010 and 2012–2021. The FCCL started each season by checking tanks housing fish from the oldest multi‐family groups (i.e., offspring from parents spawned earliest in the previous year) and checked additional multi‐family groups each week of the season as they began to reach maturity. They stopped checking for mature fish in each multi‐family group once a target number of pairs had been recovered and ended the spawning season once a total target number of pairs had been crossed (usually in May). We acknowledge the caveat that this means our dataset is artificially truncated by the imposed hatchery spawning window. Early in the season, fish might mature before hatchery managers were regularly checking the tanks (though spot checking suggests that this was uncommon; personal observations by author Luke Ellison), and late in the season, the FCCL staff ceased production before all fish reached maturity (though fish maturing after May or June was infrequent; personal observations by author Luke Ellison). Nevertheless, we can assess changes to age at maturity within the hatchery spawning season as defined by FCCL production activities. We documented the trend in age at maturity over time by relating age at maturity to spawn year, using linear regression as implemented in R 4.1.0 (R Core Team, 2022). We performed this analysis with all fish combined, as well as for regular season fish and late season fish separately.

### 2.4 Genetic component of age at maturity: Heritability

We estimated the genetic component of the trait age at maturity (i.e., phenotypic variance explained by additive genetic variance) in 2010 and then documented the change in genetic variance of the captive delta smelt population over time from 2010 to 2021 (excluding 2011). We used an animal model that is a mixed model that estimates the genetic and environmental components of phenotypic variation, using a pedigree (Kruuk, 2004). Specifically, we used a generalized linear mixed model (GLMM) using Markov chain Monte Carlo (MCMC) in the R package MCMCglmm (Hadfield, 2010) in R v. 4.0.3. These models are particularly useful for estimating genetic variance in unbalanced pedigrees, such as in wild populations (Wilson et al., 2010). We fit separate, identical model structures for each spawning year to estimate variance components and narrow‐sense heritability (*h* ^*2*^) for age at maturity over the 10‐year period. We tested the inclusion of model variables for fixed effects, such as DI and temperature regime, by estimating whether the 95% confidence intervals for the posterior distribution for the fixed variables overlap zero. We tested the inclusion of model variables for random effects including sire identity, dam identity, and Julian spawning week, using DIC score comparisons. The package MCMCglmm uses a Bayesian framework to estimate the variance contributed by each effect in the model. Thus, in our final model we set the priors to equally partition variation among all random effects. Bayesian inference allowed us to directly compare posterior probability distributions for total phenotypic and genetic variance across different years (Morrissey et al., 2014). We ran two MCMC chains in parallel for 500,000 generations with a burn‐in of 50,000 and thinning interval of 250, which we then visually checked for convergence as recommended (Wilson et al., 2010). Our autocorrelation values for the parameters were near zero, confirming that convergence occurred and there were no trends in the parameters over successive generations of the model. Heritability was calculated as the ratio of observed additive genetic variance to total phenotypic variance.

### 2.5 Genetic components of age at maturity: Domestication indices

To further investigate whether changes to age at maturity were genetically driven, we evaluated the relationship between age at maturity and DI, a measure of the number of generations an individual's genome has spent in captivity. We calculated the DI of each fish in PMx (Lacy et al., 2012), following the methods in Finger et al. (2018): wild fish have a DI of 0, and the DI of captive‐born fish is calculated as the average DI of their parents plus 1. Thus, the first generation of hatchery offspring from two wild parents would each have a DI value of 1 and their cross would produce offspring with a DI value of 2 (e.g., [[1 + 1]/2] +1 = 2). A highly domesticated fish (e.g., DI of 8.5) crossed with a low DI fish (e.g., DI of 2) would produce medium DI offspring of 6.25 (e.g., [[8.5 + 2]/2] +1 = 6.25). In the period of our study, the hatchery population of delta smelt had DIs between 0 (wild‐caught fish) and 9.63 (~9–10 generations in the hatchery), providing a diverse range of hatchery ancestries for our analyses. We evaluated the relationship between DI and age at maturity with linear regression in R 4.1.0 (R Core Team, 2022). If adaptation to captivity favored early maturation, we expected to find a negative relationship between DI and age at maturity, whereas if the trait had no genetic component or selection was not acting on this trait, we expected to find no relationship between DI and age at maturity. We performed this analysis using only regular season fish to avoid conflating variation associated with DI with variation due to temperature regime.

It is important to note that time is inherently incorporated into the variable DI, because it is a representation of the number of generations an individual's genome has been in the hatchery. Consequently, in the first few years of spawning only wild and low DI fish were available and the range of possible DI values increased each year of the spawning program. This creates a confounding effect between year and DI that prevents us from including both variables in one model. Instead, we qualitatively assessed the interactive effect of year and DI on age at maturity by plotting the median age at maturity for regular season fish binned into DI groups (1–3, 3–5, 5–7, and 7–10) for each year. If adaptation to captivity favored early maturation, we expected to observe lower median ages at maturity in higher DI groups, regardless of year. By using DI as a fixed effect in these models to look for genetic changes in the age to maturity for all spawning years combined, we can use “spawning year” to account for changing hatchery practices across years.

### 2.6 Plastic components of age at maturity

To investigate the plasticity of age at maturity, we first assessed age at maturity relative to the Julian day fish eggs were fertilized (hereafter, birthday), using only regular season fish. If age at maturity was largely heritable, we expected to observe relatively constant ages at maturity regardless of birthday. Alternatively, if age at maturity was plastic and driven more by environmental cues, we expected fish to mature during similar times of the year regardless of birthday, leading to earlier ages at maturity for fish born late in the previous season. We tested for genetic changes to plasticity by accounting for DI in this analysis. If selection was acting on the plasticity of age at maturity, we expected to find different relationships between age at maturity and Julian birthday for different DI groups. We used linear regression in R 4.1.0 (R Core Team, 2022) to relate age at maturity to Julian birthday and DI, combining data among years 2010–2021.

We also investigated the plasticity of age at maturity by evaluating patterns of when females became ripe (i.e., when sexually mature females express a large volume of developed eggs and are ready to spawn) throughout the season each year. If the age at which females ripen was largely heritable, we expected to observe relatively constant numbers of females becoming ripe throughout the season, since in the previous year fish were spawned weekly (and therefore, fish were born throughout a continuous period). If the age at which females ripen was driven more by environmental cues, however, we expected to observe pulses of large numbers of ripe females during each spawning season. We characterized the density of ripe females per week of the spawning season separately for each year 2010–2021 (excluding 2011) to evaluate patterns within and among years.

### 2.7 Fitness of age at maturity

To understand whether changes in age at maturity and plasticity of this trait are driven by domestication selection, we evaluated fitness in relation to DI. For selection to act on age at maturity in the hatchery, there needs to be a strong fitness component associated with the age of the fish. Therefore, we evaluated whether changes in the age of maturity or changes in the plasticity of age at maturity were associated with fish that have higher fitness. We were able to evaluate the fitness of parents in the hatchery by measuring their reproductive success (i.e., the number of offspring that survived to adulthood and were tagged, genotyped, and genetically assigned to a given parent). We first tested for variation in reproductive success among parent DI groups, using an analysis of variance followed by a post hoc Tukey HSD test in R 4.1.0 (R Core Team, 2022). While accounting for DI, we then regressed either the age parents became mature or the time of year (Julian week) parents became mature with their total reproductive success. Because hatchery practices artificially spread out spawning throughout a 3.5‐month period, we evaluated both the age at maturity and the time of year (Julian week) fish matured, to understand whether fish that reproduce at the beginning or the very end of the season had higher fitness. This is because the time of year that a fish becomes mature is a combination of both when in the previous season they were born and how quickly they matured. These analyses excluded fish spawned in 2009 due to a lack of data on which week parents were tagged, and they also excluded fish spawned in 2010 because their offspring were tagged irrespective of sexual maturity in 2011. Therefore, these analyses included parents spawned in 2011–2020. We performed these analyses using only regular season fish to avoid variation due to temperature regime rather than the variables of interest.

## 3 RESULTS

### 3.1 Increase in age at maturity over time

**FIGURE 1:**
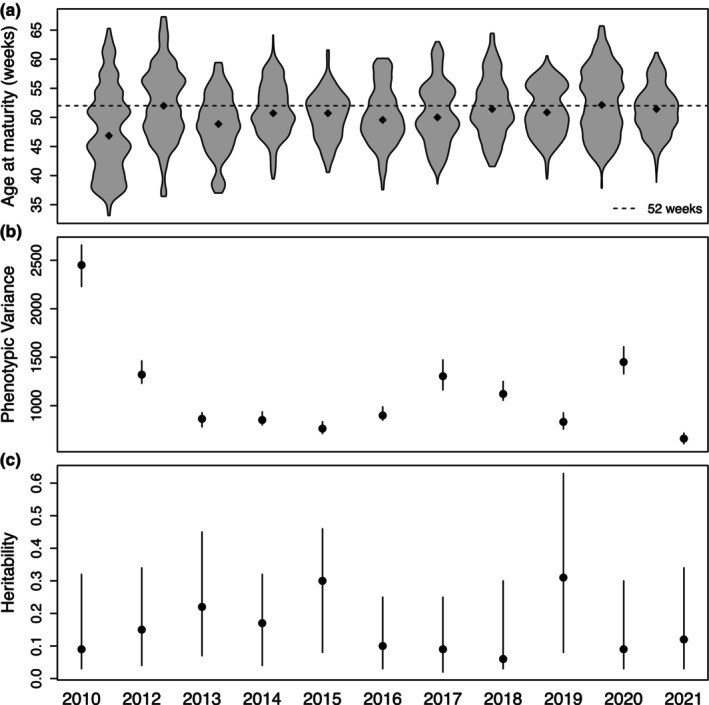
Change in (a) age at maturity, (b) phenotypic variance, and (c) heritability over time for all fish in the hatchery in 2010–2021 (excluding 2011). Dotted black line in panel (a) indicates 52 weeks (the number of weeks in 1 year) for reference. Panels (b) and (c) show point estimates with 95% confidence intervals generated by the top model in Table S2.

The overall median age at maturity in captive delta smelt was 50.6 ± 5.2 weeks (see Table S1 for statistics by year). We observed a small, but significant positive relationship between age at maturity and spawn year with an increase in age at maturity of approximately 0.2 weeks (1.4 days) per year (Figure 1a; Table 1a; *p* = 1.5e‐85), for a total increase of 2.2 weeks (15.4 days) from 2010 to 2021. This positive relationship may largely be driven by the lower range of ages at maturity in 2010, a year in which tagging started earlier than subsequent years (Figure S2). Therefore, we additionally performed a regression of age at maturity in the years 2012–2021 and found a smaller, but still significant, positive relationship with spawn year with an increase in age at maturity of approximately 0.12 weeks per year (Table 1b; *p* = 1.4e−23). When we separated regular season fish and late season fish, we observed that late season fish matured at a younger age than regular season fish (Figure S3), though the temporal trend in age at maturity for the two groups was parallel, indicating that temperature regime did not affect the shift in age at maturity over time.

**TABLE 1:** For each linear regression model, the response variable, model covariate(s), the beta coefficient for each covariate, *p*‐values for each beta coefficient, the *R* ^2^ of the model (with the *p*‐value of the model in parentheses), and a description of which dataset was used for each analysis.

	Response variable	Model covariate(s)	Beta coefficient	Beta coefficient *p*‐value	Model *R* ^2^ (*p*‐value)	Dataset used
(a)	Age at maturity	Intercept	49.59	0	0.02 (1.5e−85)	All fish; 2010–2021
		Spawn year	0.20	1.5e−85		
(b)	Age at maturity	Intercept	50.31	0	0.004 (1.4e−23)	All fish; 2012–2021
		Spawn year	0.12	1.4e−23		
(c)	Age at maturity	Intercept	50.41	0	0.006 (4.6e−30)	Regular season only; 2010–2021
		DI	0.21	4.6e−30		
(d)	Age at maturity	Intercept	61.32	0	0.15 (*p* = 0)	Regular season only; 2010–2021
		Birthday	−0.13	9.5e−180		
		DI	−0.57	1.1e−25		
		Birthday x DI	0.007	3.5e−22		
(e)	Reproductive success	Intercept	−0.73	0.91	0.04 (4.14e−32)	Regular season only; 2011–2020
		Age at maturity	0.11	0.41		
		DI	3.21	0.007		
		Age at maturity x DI	−0.04	0.09		
(f)	Reproductive success	Intercept	3.13	7.3e−05	0.04 (1.8e−31)	Regular season only; 2011–2020
		Week mature	0.03	0.62		
		DI	1.21	1.2e−32		

### 3.2 Genetic components of age at maturity: Low genetic variation and heritability

Our best fit model of the genetic component of age at maturity included temperature regime as a fixed effect and dam identity as a random effect (Table S2). We calculated the amount of phenotypic variation (and associated components, such as additive genetic variation and variation due to dam identity) present for age at maturity in the hatchery population from 2010 to 2021. We found that phenotypic variation was the highest in 2010, sharply declined in 2012 and 2013, and then remained relatively constant and low until 2021 (Figure 1b; Table S3). Despite high phenotypic variation observed in 2010, very little of this variation was explained by genetic variation, and heritability estimates were low (~0.09). The high phenotypic variation observed in 2010 is most likely driven by the larger range of ages at maturity for this year, where tagging started earlier than subsequent years (Figure S2). Heritability did not significantly change between 2010 and 2021 (Figure 1c), with an overall mean heritability of 0.15 (Table S3).

### 3.3 Genetic components of age at maturity: Older age at maturity for higher DI fish

**FIGURE 2:**
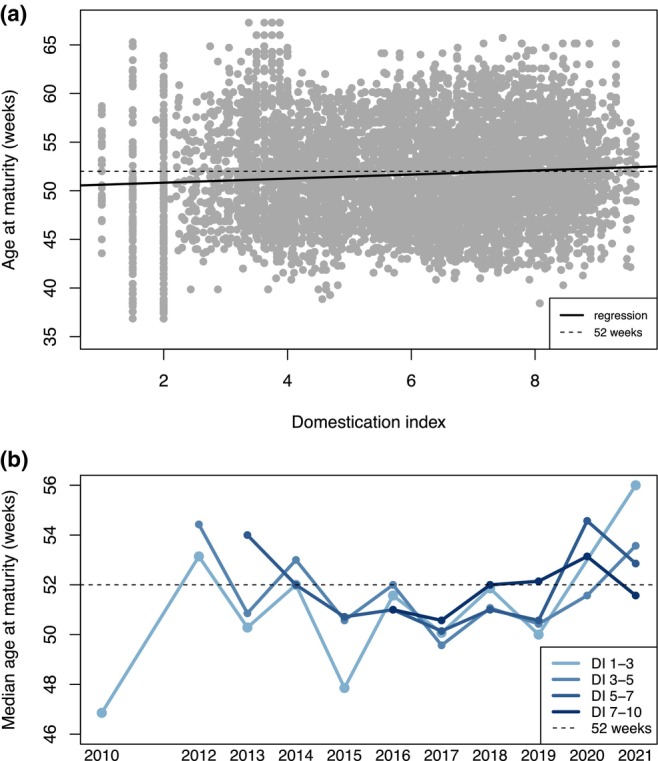
Age at maturity relative to domestication index (DI) for regular season fish (i.e., excluding late season fish that experienced a different temperature regime) in 2010–2021 (excluding 2011). Panel (a) shows a linear regression of age at maturity and DI, with all years combined. Panel (b) shows the median age at maturity for four DI groups over time, with sample sizes for median calculations in Table S4. The dotted black line in both panels indicates 52 weeks (the number of weeks in 1 year) for reference.

Age at maturity positively correlated with DI, with an increase in age at maturity of approximately 0.21 weeks for each 1 unit increase in DI (Figure 2a; Table 1c, *p* = 4.6e−30). In contrast to the predicted negative relationship that could arise from domestication selection favoring early maturation, we observed a positive relationship where higher DI fish took longer to mature. While the dominant trend shows that higher DI fish mature more slowly, we also qualitatively observed that this trend fluctuated through time (Figure 2b). Only low DI fish existed in the early years of the program, whereas high DI fish only appeared in 2016, and there were far fewer low DI fish in later years of the program (Figure 2b; Table S4). Consequently, the increase in age at maturity over time may be explained by the higher abundances of high DI fish that have older ages at maturity.

### 3.4 Plastic components of age at maturity: Fish born later in the year mature faster

**FIGURE 3:**
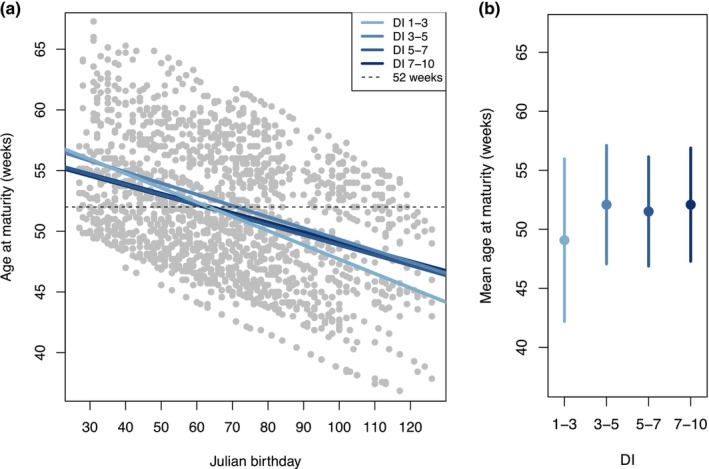
Age at maturity relative to Julian birthday (a). Data are combined across the years 2010–2021 (excluding 2011) for regular season fish (i.e., excluding late season fish that experienced a different temperature regime), and regression lines are shown for each DI group. Dotted black line indicates 52 weeks (the number of weeks in 1 year) for reference. Panel (b) shows the mean age at maturity for each DI group, with error bars representing standard deviation.

Julian birthday significantly affected age at maturity, where fish born later in the year matured at a faster rate. For example, the median age at maturity for fish born in January was 52 weeks, whereas the median age at maturity for fish born in May was 45 weeks. For each Julian day later in the year that a fish was born, they matured more quickly by approximately 0.13 weeks (i.e., ~1 day; *p* = 9.5e−180). We also found evidence for a genotype‐by‐environment interaction, where the effect of Julian birthday on age at maturity depended on DI (significant DI‐by‐Julian birthday interaction; Figure 3a; Table 1d). This interaction influenced the slope of each DI groups relationship between age at maturity and birthday, leading to lower plasticity (i.e., shallower slope) of age at maturity in medium and high DI fish (Figure 3a). We also observed reduced variation in age at maturity for fish with higher DIs (3–10) compared to the lowest DI group (1–3), providing further evidence for less plasticity in higher DI fish (Figure 3b).

### 3.5 Plastic components of age at maturity: Synchronous maturation

**FIGURE 4:**
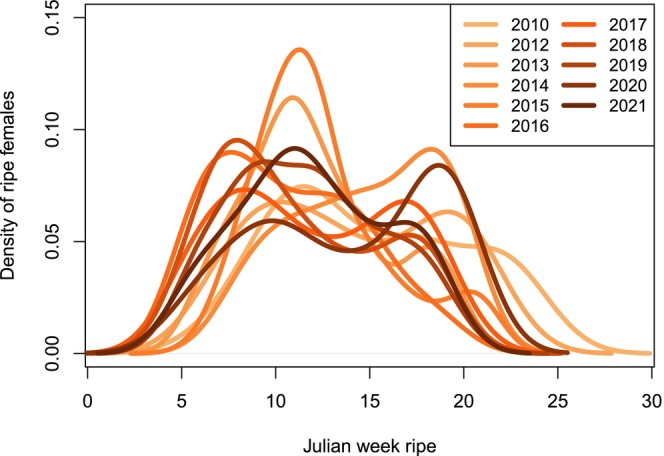
Density of females that became ripe in the hatchery each week of the spawning season, in each year 2010–2021 (excluding 2011).

The number of females becoming ripe each week varied throughout the season, with the highest number of females becoming ripe in March–April. If this trait was under tight genetic control, we would expect the number of females becoming ripe each week to be consistent throughout the season since hatchery practices spawn roughly the same number of fish each week. Instead, most females became ripe at approximately the same time in the season, with one or two pulses of large numbers of ripe females each year (Figure 4), suggesting some plastic control of the trait (i.e., fish born later in the season must mature more quickly to become ripe at the same time as fish born earlier in the season).

### 3.6 Selection pressures for age at maturity: Higher fitness of high DI genotypes

**FIGURE 5:**
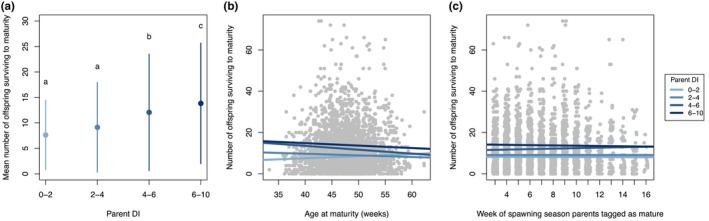
The mean number of offspring surviving to maturity (a measure of reproductive success) for parents in each DI group (a). We related the number of surviving offspring to each parent's (b) age at maturity and (c) week they were tagged as sexually mature, accounting for DI group in each analysis. Data are combined across the years 2010–2021 (excluding 2011) for regular season fish (i.e., excluding late season fish that experienced a different temperature regime), and regression lines are shown for each DI group.

We confirmed the pattern observed by Finger et al. (2018) that more offspring survive to sexual maturity from crosses between higher DI parents than lower DI parents (Figure 5). Our post hoc Tukey's test indicated no significant difference in fitness for parents in the two lower DI groups (0–2 and 2–4), but the DI 4–6 group had significantly higher fitness than the two lower groups, and the DI 6–10 group had significantly higher fitness than any other DI groups (Figure 5a). High DI fish produced on average 6 more offspring (81% higher reproductive success) than low DI fish (Figure 5a). When we evaluated whether this fitness differential related to maturation timing, we found that the number of offspring surviving to sexual maturity was not affected by the age at which parents became sexually mature (Figure 5b; Table 1e; *p* = 0.41), nor by when in the season parents became sexually mature (Figure 5c; Table 1f; *p* = 0.62). However, in both of these models, we also confirmed that DI was the most significant variable correlated to fitness.

## 4 DISCUSSION

Adaptation to captivity can occur as quickly as the first generation of captive spawning (Lorenzen et al., 2012), which is why genetically informed breeding was an integral component of the original management plan for the FCCL delta smelt refuge population. Instead of randomly selecting broodstock for mating (Fisch et al., 2015) or allowing fish to spawn freely in the hatchery (LaCava et al., 2015), spawning pairs of delta smelt were carefully selected to minimize inbreeding, equalize family sizes, and include wild genotypes (Fisch et al., 2013). Despite these efforts, delta smelt have shown evidence of adaptation to captivity: fish with higher DI (higher hatchery ancestry) had higher reproductive success in the hatchery (Finger et al., 2018). We observed that this pattern has continued in more recent years (Figure 5). Because the timing of sexual maturation can influence access to reproductive opportunities, selection acting on age at maturity in the hatchery is one possible mechanism for the observed fitness disparity. We investigated whether there have been changes to age at maturity in delta smelt since the implementation of the spawning program and whether genetic or plastic components explain changes to this trait over time.

### 4.1 Slower maturation rates over time in hatchery population

We observed a subtle trend toward slower maturing fish by ~0.2 weeks per year from 2010 to 2021 for a total increase of 2.2 weeks, suggesting that fish reached sexual maturity at an older age over time. Domestication selection has been identified as a mechanism driving faster rates of maturity in captive salmonid spawning programs and consequently reduced fitness in the wild (Ford et al., 2012; Tillotson et al., 2019). While hatchery and genetic management of delta smelt have been successful at preventing early maturation, we unexpectedly found that late maturing individuals have become more common. We also found that this trend is likely driven by higher DI fish. We found that higher DI fish were slightly older when they reached maturity (Figure 2); however, we did not find a corresponding fitness differential to suggest domestication selection was acting in the hatchery. For example, we did not observe higher fitness for fish maturing at older ages (Figure 5b), as would be expected if this trait was under directional selection in the hatchery. Instead, we hypothesize that this trend is driven by a loss of plasticity in highly domesticated fish as a result of relaxed selection pressures in the hatchery. Below, we provide evidence for this hypothesis, including evidence of low heritability, plasticity in the trait, and a loss of plasticity in higher DI fish.

### 4.2 Low heritability for age at maturity in delta smelt

We assessed the potential for adaptation to the hatchery environment by estimating the genetic component (i.e., heritability) of variation in age at maturity between 2010 and 2021. We found that the mean heritability for age at maturity in the hatchery was 0.15, suggesting that this trait is partially under genetic control, but is largely influenced by environmental factors and phenotypic plasticity. A review of salmonid studies found that heritability of age at maturity had a range of 0–0.75 (median = 0.21) based mostly on data from hatchery and farmed fish (Carlson & Seamons, 2008), and a study on hatchery‐born wild‐reared steelhead found spawning day to have a heritability of 0.5 (Abadía‐Cardoso et al., 2013). High heritability for age at maturity means that this trait could be quickly influenced by natural selection in salmonid hatcheries, and previous research has demonstrated domestication selection favoring early maturation in a Chinook salmon hatchery (Ford et al., 2012). Large changes in a single trait over time by selection are more likely with high heritability and high selection pressure (Araki et al., 2008). We reaffirmed findings by Finger et al. (2018) that higher DI fish had higher fitness in the hatchery, but we found no fitness advantage associated with early or late maturation (Figure 5), suggesting that direct selection on age at maturity is likely not acting in this system. Instead, the low heritability of the trait and lack of a selection (i.e., fitness) differential between early and late maturing fish suggests age at maturity may largely be controlled by phenotypic plasticity.

### 4.3 Age at maturity is a plastic trait relative to when fish are born in the season

In the hatchery where environmental conditions such as diet and population density were kept consistent, and when we accounted for temperature regime, we found that age at maturity was primarily controlled by plasticity, depending on when in the season fish were born. We hypothesize that plasticity for age at maturity likely originated in wild individuals of delta smelt and has been retained in the hatchery. Age at maturity has been demonstrated to be a plastic trait under different thermal regimes (Kuparinen et al., 2011) or different diets (Larsen et al., 2006), with warmer rearing temperatures or larger food rations promoting faster development and thus younger ages at maturity. In delta smelt, given that fish born later in the year matured faster and we observed peaks in when females became ripe each season, we hypothesize that the selective pressure maintaining plasticity for age at maturity may be attributed to potential fitness benefits of synchronous maturation. Synchronous maturation may improve chances of finding a mate, increase fertilization success, and/or dilute predation rates in the wild (Molloy et al., 2012). However, it is unknown what the direct selective pressure may be for synchronous spawning in the wild for delta smelt. Phenotypic plasticity of any trait depends on reliable environmental cues and machinery to sense those cues (Getty, 1996). Given that most environmental conditions are kept consistent in the hatchery, an environmental cue such as day length or lunar cycles could contribute to when fish become sexually mature (Takemura et al., 2010), though one study found no evidence of lunar cycles influencing delta smelt spawning migration in the wild (Bennett & Burau, 2015). Alternatively, synchronous maturation could arise from intraspecific chemical cues (Sorensen & Stacey, 1999). Captive delta smelt are housed in separate tanks organized by multi‐family groups, but the FCCL facility uses recirculating water systems that could allow for biochemical exchange among tanks (Huertas et al., 2006; Lindberg et al., 2013). Experimental evaluation of the role of photoperiod or chemical cues could help elucidate the environmental driver(s) contributing to plastic control of age at maturity and female ripening. Identifying environmental cues for maturation could provide the hatchery a tool for managing the spawning season, for example, by deliberately cueing fish to mature in aggregate. Measuring growth and the size of delta smelt at age at maturity may also elucidate possible mechanisms of plasticity (Mobley et al., 2020, 2021). Captured delta smelt individuals have been shown to have longer fork lengths and higher body conditions than wild‐caught individuals at the time they are spawned (Ellison et al., 2023), but growth indices were not collected when fish were tagged as mature (fish are not usually spawned as soon as they are mature, thus allowing more time for growth before they are spawned). Further research could investigate whether age at maturity is correlated with growth indices among different DI groups. For example, if higher DI fish mature more slowly, they may be larger at the time they reach sexual maturity, possibly increasing their fitness (Mobley et al., 2020).

### 4.4 Relaxed selection may explain a loss of phenotypic plasticity for age at maturity

In addition to documenting plasticity in age at maturity, we observed variation in the extent of plasticity among different DI genotypes. This genotype‐by‐environment interaction (i.e., slope of the reaction norms, Figure 3a) suggests that evolution may be acting on the plasticity for age at maturity (Gutteling et al., 2007; Windig et al., 2004). Fish of medium and high DI had reduced plasticity in their age at maturity, which resulted in higher DI fish born late in the season maturing slower than lower DI fish born late in the season. In the wild, it is most likely adaptive for an individual to be able to modify its rate of maturity to synchronize with mass spawning events, and this aligns with the steeper reaction norm (i.e., higher plasticity) we observed in low DI fish (Figure 3). We hypothesize that the change in plasticity for age at maturity in high DI fish is not due to directional selection (i.e., trait expression favored by selection, commonly referred to as genetic accommodation; West‐Eberhard, 2003; Baldwin, 1902), given that we observed no fitness differences related to age at maturity or when fish matured throughout the season (Figure 4). Instead, we hypothesize that these differences in plasticity may be the result of hatchery spawning practices that have relaxed wild selection pressures and thereby removed the negative fitness consequences of maturing too late in the season. Hatchery staff spawned approximately the same number of fish each week throughout a long ~3.5‐month season, consequently allowing late‐born and slow maturing fish to have reproductive opportunities at the end of the following spawning season. We also found evidence that this relaxation of selection pressure that leads to a loss of phenotypic plasticity may occur relatively quickly in the hatchery (i.e., the first few generations of captivity), given that both medium and high DI fish had similar reaction norms for maturation timing as compared to the reaction norm for low DI fish (Figure 3a).

The ability to accurately assess an individual's current environment and appropriately change its phenotype (i.e., phenotypic plasticity) can increase an individual's fitness. However, theory on the evolution of plasticity suggests that it is costly to maintain and there may be associated tradeoffs (Dewitt et al., 1998; Murren et al., 2015). This has been demonstrated in controlled experiments where the less plastic genotype has higher fitness in the absence of the selective pressure (Agrawal et al., 2002) and in wild populations where a loss of phenotypic plasticity is observed when environments are stable over multiple generations (i.e., genetic assimilation; Waddington, 1959; Oostra et al., 2014; Snell‐Rood et al., 2010). This loss of plasticity, also known as canalization, can occur through changes in among‐individual variance of a trait (age at maturity in our case) due to individual differences in developmental plasticity (Crispo, 2007; West‐Eberhard, 2003). We observed a reduction in among‐individual variation in age at maturity for high DI fish compared to low DI fish (Figure 3b).

In the delta smelt hatchery, low DI genotypes had higher plasticity and lower fitness (i.e., fewer number of surviving offspring) than higher DI genotypes which have lower plasticity but higher fitness. This fitness differential is maintained regardless of when fish are born in the season (i.e., no observed selection pressure for when fish are born; Figure 5). Therefore, we hypothesize that the loss of plasticity in high DI fish may be adaptive in that they can direct energy toward other processes (such as growth and reproduction) rather than energy required for plasticity maintenance. Indeed, higher DI fish are larger and have a higher body conditioning (Ellison et al., 2023). While there are likely many selective pressures in the hatchery that have contributed to the observed higher fitness of high DI genotypes, one possible contribution could come from the lower fitness costs related to plasticity maintenance.

### 4.5 Conservation implications

Overall, we conclude that the increase in age at maturity in the delta smelt hatchery over time is likely due to older ages at maturity for high DI fish that are more abundant in 2020–2021. However, the low heritability and lack of a fitness differential between early and late maturing fish suggests that this trait is most likely not under domestication selection in the hatchery. Rather, we found that this trait was strongly influenced by plasticity related to Julian birthday, where fish born later in the year matured faster, potentially to ensure reproductive success when most fish were ready to spawn. However, we found a loss of phenotypic plasticity with increasing DI. We propose that relaxed selection pressure in the hatchery environment, possibly due to hatchery managers spawning fish outside of pulses when many females become ripe, may allow slower maturation in late‐born fish. If this is the case, when slower maturing fish return to the wild they might have lower fitness if they cannot rapidly mature to synchronize their gonadal development with peak population spawning periods. Further research is needed to understand whether this plastic response can be regained by medium and high DI fish if they return to the wild, but if not, re‐introduced fish may not be as responsive to environmental cues to mature and this could affect access to reproductive opportunities.

After more than ten generations of captive spawning to maintain the refuge population, captive‐born delta smelt are now being used to supplement the dwindling wild population (U.S. Fish and Wildlife Service, 2019). Understanding long‐term patterns of adaptation to captivity in the captive population is therefore more important now than ever. Releasing captive‐born fish into the wild will provide opportunities to investigate the relative fitness of fish with different DIs. And once captive‐born fish begin reproducing in the wild and future generations of wild‐born fish with captive ancestry are available for incorporation into the hatchery, future studies can investigate how age at maturity is affected by selection pressures in the wild.

In recent years, fish surveys have found few or no wild delta smelt to incorporate into the captive population, indicating that the genetic diversity held in the hatchery may be all that remains for the species (U.S. Fish and Wildlife Service, 2019). Continuing to genetically manage the delta smelt hatchery to maintain genetic diversity in traits like age at maturity will give fish the best chance of responding to selection pressures in the wild through plastic or genetic changes. Further studies to investigate other mechanisms of adaptation to captivity would continue to help elucidate why higher DI fish experience higher fitness in the hatchery and how to limit further genetic change.

## Supporting information


Data S1.

## Data Availability

Data and scripts for the analyses and generation of figures in this manuscript are available at https://github.com/meflacava/DS‐MaturationTiming.
