# Supplementary material for: Loss of plasticity in maturation timing after ten years of captive spawning in a delta smelt conservation hatchery
Source: Evol Appl. 2023 Nov 2;16(11):1845–57. doi: 10.1111/eva.13611 (PMC10681455; doi:10.1111/eva.13611)

Supplementary material for “Loss of plasticity in maturation timing after ten years of captive spawning in a delta smelt conservation hatchery”

**Table S1:** For each spawn year, the median, standard deviation, and sample size for age at maturity (number of weeks from fertilization to the expression of gametes).

| Year | Median | Standard deviation | Sample size |
|---|---|---|---|
| 2010 | 46.86 | 7.24 | 1352 |
| 2012 | 52 | 6.30 | 2049 |
| 2013 | 48.86 | 5.10 | 2037 |
| 2014 | 50.71 | 4.48 | 2234 |
| 2015 | 50.71 | 3.98 | 1875 |
| 2016 | 49.57 | 4.91 | 2430 |
| 2017 | 50 | 5.18 | 2687 |
| 2018 | 51.43 | 5.10 | 2307 |
| 2019 | 50.86 | 4.16 | 2327 |
| 2020 | 52.14 | 5.75 | 2216 |
| 2021 | 51.43 | 3.99 | 2912 |
| *All* | *50.57* | *5.23* | *24426* |

**Tables S2:** Models with random effects were evaluated with DIC (Deviance Information Criterion) values for fish maturing in years 2010 and 2020. The models with lowest DIC were selected for inclusion of random effects. Models with corresponding fixed effects were evaluated with posterior distributions (PD). Effects that with 95% confidence intervals overlapping zero were not included in the final model. AgeAtMaturity = number of days until male or female fish is identified as sexually mature; damID = female parent; sireID = male parent; SW = Julian spawning week of the year; animal = relatedness between individual larvae; DI = domestication index of offspring; Temp = temperature regime (regular or late season). Final models marked with *.

| **Model** | **Random Intercept** | **DIC (2010)** | **DIC (2020)** |
|---|---|---|---|
| AgeAtMaturity ~ 1<br>random ~ animal + damID | Animal, damID | 17906.83* | 23569.37* |
| AgeAtMaturity ~ 1<br>random ~ animal + damID + sireID | Animal, damID, sireID | 17935.58 | 23599.34 |
| AgeAtMaturity ~ 1<br>random ~ animal + damID + SW | Animal, damID, SW | 17940.25 | 23580.48 |

| **Model** | **Fixed Effect** | **PD (2010)** | **PD (2020)** |
|---|---|---|---|
| AgeAtMaturity ~ DI<br>random ~ animal + damID | DI | 80.2<br>(75 – 86) † | 8.6303<br>(-0.5 – 18.2) |
| AgeAtMaturity ~ Temp<br>random ~ animal + damID | Temp | -6.5<br>(-15.8 – 6.1) | -44.8*<br>(-59.8 – -31.3) |

†2010 was not a good representation for testing the inclusion of DI fixed effects because only low DI fish were present in 2010.

**Table S3:** All variance components (in weeks) for offspring age estimated from animal models (age at maturity in 2010 to 2021). N: number of offspring in animal model. $V_A$: additive genetic variance. $V_P$: Total phenotypic variance. $V_{DamID}$: Variation due to dam identity. In parentheses are 95% confidence intervals. All variance components are reported as modes from 1800 iterations of the model.

| Year | N | $V_A$ | $V_{DamID}$ | $V_P$ | Heritability |
|---|---|---|---|---|---|
| 2010 | 1718 | 807.4<br>(263.8 – 1236) | 180.1<br>(74.7 – 429.7) | 2470<br>(2286 - 2715) | 0.31<br>(0.11 – 0.47) |
| 2012 | 2050 | 1108<br>(732.3 – 1551) | 129.7<br>(50.2 – 295.8) | 1697<br>(1577 - 1945) | 0.69<br>(0.46 0.84) |
| 2013 | 2037 | 706.2<br>(260.8 – 1072.7) | 251.9<br>(95.0 – 500.0) | 1208<br>(1093 – 1353) | 0.68<br>(0.22 – 0.86) |
| 2014 | 2236 | 325.3<br>(90.9 – 570.2) | 87.2<br>(35.0 – 229.8) | 961.2<br>(914.3 – 1079) | 0.33<br>(0.10 – 0.55) |
| 2015 | 1876 | 219.5<br>(51.5 – 375.5) | 69.35<br>(29.0 – 174.5) | 775.0<br>(721.4 – 854.4) | 0.33<br>(0.06 – 0.46) |
| 2016 | 2521 | 243.3<br>(51.7 – 501.3) | 238.8<br>(100.8 – 352.8) | 1065<br>(981.3 – 1170) | 0.17<br>(0.04 – 0.45) |
| 2017 | 2857 | 113.9<br>(31.5 – 357.2) | 645.1<br>(488.4 – 850.8) | 1417<br>(1258 – 1601) | 0.06<br>(0.02 – 0.25) |
| 2018 | 2416 | 108.1<br>(24.7 – 398.7) | 410.0<br>(262.3 – 542.8) | 1303<br>(1201 – 1437) | 0.08<br>(0.02 – 0.30) |
| 2019 | 2414 | 569.3<br>(115.2 – 789.5) | 106.0<br>(27.0 – 305.6) | 899.0<br>(837.1 – 1048) | 0.66<br>(0.15 – 0.81) |
| 2020 | 2406 | 121.0<br>(37.3 – 589.9) | 529.7<br>(309.7 701.9) | 1611<br>(1490 – 1827) | 0.07<br>(0.03 – 0.36) |
| 2021 | 2912 | 38.9<br>(8.6 – 153.2) | 161.1<br>(95.0 – 204.3) | 438.0<br>(397.4 – 485.0) | 0.07<br>(0.03 – 0.36) |

**Table S4:** Sample sizes for calculating median age at maturity for each DI group in each spawn year, using only regular season fish.

| | Sample sizes for calculating median | | | |
|---|---|---|---|---|
| **Year** | **DI 1-3** | **DI 3-5** | **DI 5-7** | **DI 7-10** |
| 2010 | 1106 | 0 | 0 | 0 |
| 2012 | 117 | 1396 | 0 | 0 |
| 2013 | 30 | 1427 | 17 | 0 |
| 2014 | 22 | 270 | 1235 | 0 |
| 2015 | 51 | 415 | 1231 | 0 |
| 2016 | 27 | 113 | 1264 | 379 |
| 2017 | 56 | 294 | 992 | 1079 |
| 2018 | 85 | 290 | 465 | 1110 |
| 2019 | 37 | 106 | 684 | 1272 |
| 2020 | 1* | 142 | 566 | 1364 |
| 2021 | 19 | 311 | 254 | 1688 |

*excluded median for n=1

**Figure S1:** Temperature regimes experienced by regular season fish (orange) and late season fish (green) over the course of their ~1 year life span. Eggs are incubated at 16.5˚C and juvenile are reared at this temperature until late fall when the temperature is lowered to 12˚C to reduce stress from handling during tank transfers. Regular season fish (orange) are kept at 12˚C until spawning, while late season fish (green) have temperature increased back to 16.5˚C after handling for as long as possible before lowering back to 12˚C for spawning. Both groups experience the higher temperature (16.5˚C) for approximately the same total length of time, however the regular season fish experience the higher temperature for one continuous time period, while the late season fish experience the higher temperature in a discontinuous manner.

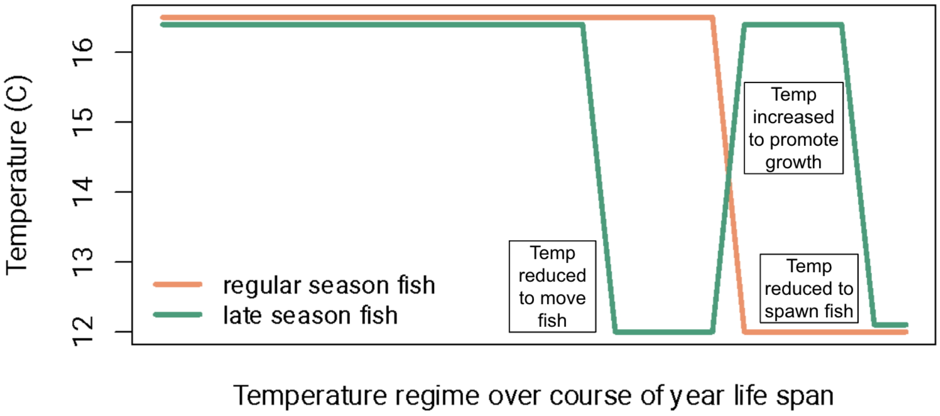


**Figure S2:** Range of dates fish were tagged as sexually mature during each spawning season in 2010–2021.

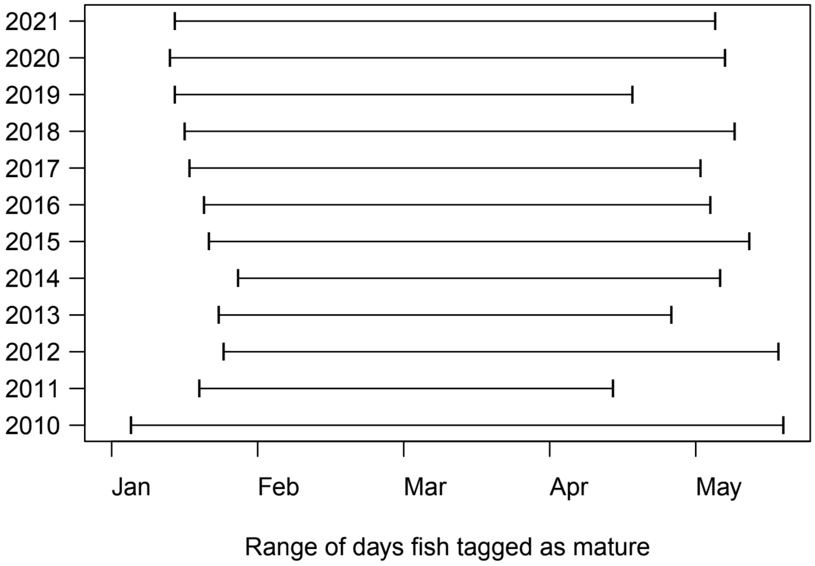

**Figure S3:** Age at maturity by year for fish maturing in 2010–2021, separated by temperature regime (regular season versus late season; see Figure S1). Points are jittered within year to increase visibility. Each line represents the linear regression for the color-coordinated temperature regime.

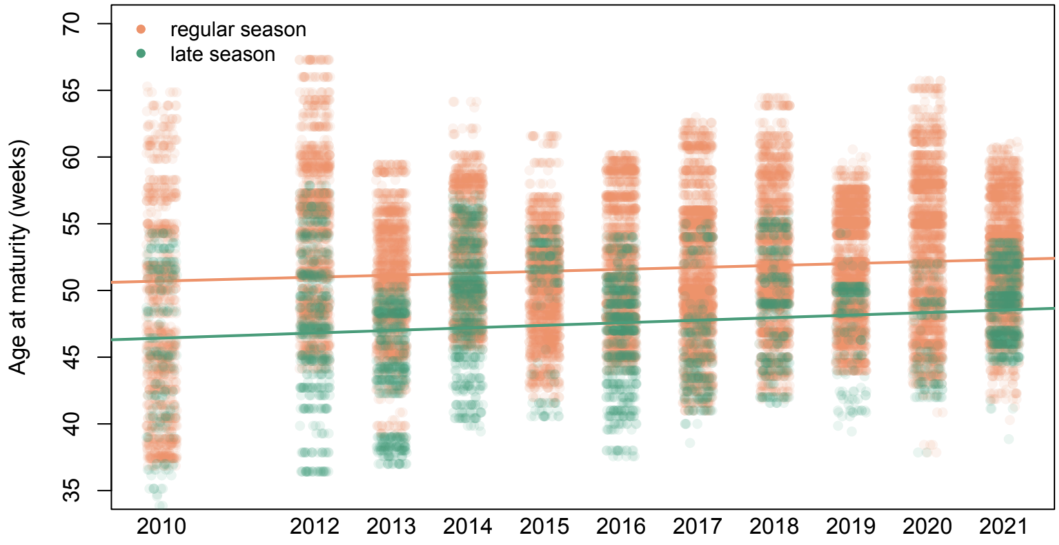

Supplement: Supplementary file 1 — Data S1. [file EVA-16-1845-s001.pdf]
